# The Recognition of Calmodulin to the Target Sequence of Calcineurin—A Novel Binding Mode

**DOI:** 10.3390/molecules22101584

**Published:** 2017-09-21

**Authors:** Chia-Lin Chyan, Deli Irene, Sin-Mao Lin

**Affiliations:** Department of Chemistry, National Dong Hwa University, Hualien 974, Taiwan; deli24@gate.sinica.edu.tw (D.I.); 610312003@gms.ndhu.edu.tw (S.-M.L.)

**Keywords:** calmodulin, calcineurin, NMR, protein structure

## Abstract

Calcineurin (CaN) is a Ca^2+^/calmodulin-dependent Ser/Thr protein phosphatase, which plays essential roles in many cellular and developmental processes. CaN comprises two subunits, a catalytic subunit (CaN-A, 60 kDa) and a regulatory subunit (CaN-B, 19 kDa). CaN-A tightly binds to CaN-B in the presence of minimal levels of Ca^2+^, but the enzyme is inactive until activated by CaM. Upon binding to CaM, CaN then undergoes a conformational rearrangement, the auto inhibitory domain is displaced and thus allows for full activity. In order to elucidate the regulatory role of CaM in the activation processes of CaN, we used NMR spectroscopy to determine the structure of the complex of CaM and the target peptide of CaN (CaNp). The CaM/CaNp complex shows a compact ellipsoidal shape with 8 α-helices of CaM wrapping around the CaNp helix. The RMSD of backbone and heavy atoms of twenty lowest energy structures of CaM/CaNp complex are 0.66 and 1.14 Å, respectively. The structure of CaM/CaNp complex can be classified as a novel binding mode family 1–18 with major anchor residues Ile^396^ and Leu^413^ to allocate the largest space between two domains of CaM. The relative orientation of CaNp to CaM is similar to the CaMKK peptide in the 1–16 binding mode with N- and C-terminal hydrophobic anchors of target sequence engulfed in the hydrophobic pockets of the N- and C-domain of CaM, respectively. In the light of the structural model of CaM/CaNp complex reported here, we provide new insight in the activation processes of CaN by CaM. We propose that the hydrophobic interactions between the Ca^2+^-saturated C-domain and C-terminal half of the target sequence provide driving forces for the initial recognition. Subsequent folding in the target sequence and structural readjustments in CaM enhance the formation of the complex and affinity to calcium. The electrostatic repulsion between CaM/CaNp complex and AID may result in the displacement of AID from active site for full activity.

## 1. Introduction

Calmodulin (CaM) is a highly conserved 17 kDa eukaryotic Ca^2+^-binding protein. In response to a Ca^2+^ signal, CaM interacts with and regulates various proteins including calmodulin-dependent protein kinases and phosphatases, cytoskeletal proteins, ion channels, and pumps. Unraveling its diverse roles in activation mechanisms and target recognition has received extensive attention [1,2,3,4,5,6,7]. CaM contains two globular domains connected by a flexible linker [8,9]. Each domain contains two EF-hand Ca^2+^-binding motifs. Although the two domains share high degree of homology in sequence, they exhibit subtle differences in structures and Ca^2+^ affinity [10,11,12]. Ca^2+^ influx triggers conformational changes of CaM resulting in the opening of the hydrophobic pockets allowing the association with various target proteins. Therefore, the interplay of Ca^2+^-concentration and affinity between CaM and target proteins controls and tunes CaM's diverse recognition network. Since high resolution structures of the CaM in complex with its target enzymes are mostly unavailable to date, the three-dimensional structures of the complex of CaM and various CaM-binding domains of its target enzymes have been used as mimetic structure models in the past [13,14,15,16,17,18,19,20,21,22,23,24,25,26,27,28,29,30,31,32,33,34,35,36,37,38,39,40,41]. It has been shown that CaM adopted virtually identical backbone structures upon association with either CaM-dependent enzyme or its CaM-binding domain, as judged by backbone chemical shift changes in solution [42]. Hence the CaM-binding domains in the form of peptides may serve as structure surrogates in the study of the CaM-target protein interactions. Although little sequence homology has been found in the canonical target sequences, the positions of several bulky hydrophobic residues are often conserved, allowing further classification of target sequences into recognition motifs. Among the solved structures in the 1:1 stoichiometry class, several CaM binding modes have been classified by the spacing between conserved hydrophobic anchor residues in the target peptide, which forms an α-helical structure. The widely adopted structural model is that the α-helical target peptide lies in a hydrophobic channel formed by the two domains of CaM. The predominant interactions included mostly hydrophobic forces between the anchor residues of the target peptide and two hydrophobic pockets provided by CaM. The 1–14 CaM-target binding mode, the prototype for CaM-target interactions in the 1:1 stoichiometry, was first defined in the structures of CaM bound to the M13 peptide derived from the skeletal muscle myosin light chain kinase. The hydrophobic anchor, Trp 1 and Phe 14 of M13 was engulfed in the hydrophobic pocket of the C- and N-terminal domain of CaM, respectively [13,14]. Subsequently, the 1–7, 1–10, 1–16, and 1–18 binding types were found in NMDA receptor NR1 subunit [28], CaMKII [15,16], CaMKK [17,19], and Ca^2+^ pump [32]. In most of the solved structures, the relative orientation of the target peptide to CaM are similar to the CaM/M13 peptide complex, with N- and C-terminal hydrophobic anchors of target sequence engulfed in the hydrophobic pockets of the C- and N-domain of CaM, respectively. However, the orientation of the α-helix of CaMKK is opposite to that of the other target sequences with respect to the two domains of CaM. In addition to the 1:1 binding class, two more classes of CaM/CaM-binding domain complex have been identified by the 1:2, and 2:2 (CaM:CaM-binding peptide) stoichiometric ratios [2,3,20,22,43,44]. The case of CaM, a central coordinator for many target proteins clearly indicates that the conformation of CaM depends on its interacting partners and environment. It is of great interest to determine the crucial residues in these target sequences that are responsible for driving the interaction with CaM, as the information may assist in mapping the entire network of CaM-mediated pathways. Hence, together with the proteomic and structural genomic analyses on protein interactions, the entire complement of protein structure-function relationships will take shape.

Calcineurin (CaN) is a unique Ca^2+^/calmodulin-dependent Ser/Thr protein phosphatase. CaN directs the flow of information of calcium signals to downstream effectors that controls cellular responses and gene expressions [45,46,47,48,49,50]. CaN comprise two subunits, a catalytic subunit (CaN-A, 60 kDa) and a regulatory subunit (CaN-B, 19 kDa). The sequences are highly conserved among different organisms, although there are three isoforms of CaN-A, and two isoforms of CaN-B in human. CaN-A α-isoform is composed of the catalytic domain (residue 1–348) and the regulatory domain (residue 349–521) comprising of the CaN-B binding domain (BBD; residue 348–369), CaM target domain (residue 391–414) and auto-inhibitory domain (AID; residue 468–491) [51,52]. CaN-A tightly binds to CaN-B in the presence of minimal level of Ca^2+^, but the enzyme is inactive until activated by CaM. The activation of CaN by CaM including several sequential steps was proposed more than two decades ago [53,54]. The conformational change of CaN is triggered by occupancy of calcium ions to the low affinity sites of CaN-B subunit which causes the dissociation of the CaM target domain from the CaN-B binding domain. Subsequently, CaM recognizes this target region and leads to the displacement of AID from the active site and full activation. Although this activation model is in accord with most biochemical results, there is still limited structural information. The crystallographic structure of CaN shows that the AID adopts a helical conformation and blocks the active site of CaN (PDB 1AUI), whereas the regulatory domain of CaN-A is invisible in the electron density map except AID and BBD [55]. Although many research groups, including us, have aimed to put back the missing pieces for the activation action of CaM on CaN [56,57,58], there is no high resolution structure solved for the entire CaN and CaM complex till now. Instead, two groups solved the crystal structures of CaM and the target peptide of CaN for structural surrogates [59,60]. The crystallographic structures (2R28 and 2W73) show 2:2 domain swapping structures, in which the N-domain of one CaM molecule and C-domain of a second CaM molecule interact with a target peptide of CaN, the rest C- and N-domain of the two CaM molecules interact with a second target peptide of CaN. While majority of the evidences by us and other groups support the 1:1 wrapped around structure under physiological conditions [61,62,63], no solution structure of CaM and the target peptide of CaN has been obtained. A crystallographic structure of CaM with the target peptide of CaN in 1:1 stoichiometric ratio was recently solved in 1.9 Å resolution [41]. Our solution phase structure further verifies their conclusion arrived at by x-ray crystallography, a methodology that sometimes produces structural artefact due to crystal lattice packing. Here, we report the solution structure of CaM and the target sequence of CaN (residue 391–414 in CaN-A; CaNp) by NMR. In light of the complex structure reported here, we also proposed new insight in the recognition and activation processes of CaN by CaM. We suggested that the interplay of hydrophobic and ionic interactions controls and tunes the recognition and activation processes of CaN by CaM.

## 2. Results and Discussion

### 2.1. Stoichiometry and Secondary Structure Analysis of CaNp upon Binding to CaM by Circular Dichroism Spectroscopy

**Figure 1:**
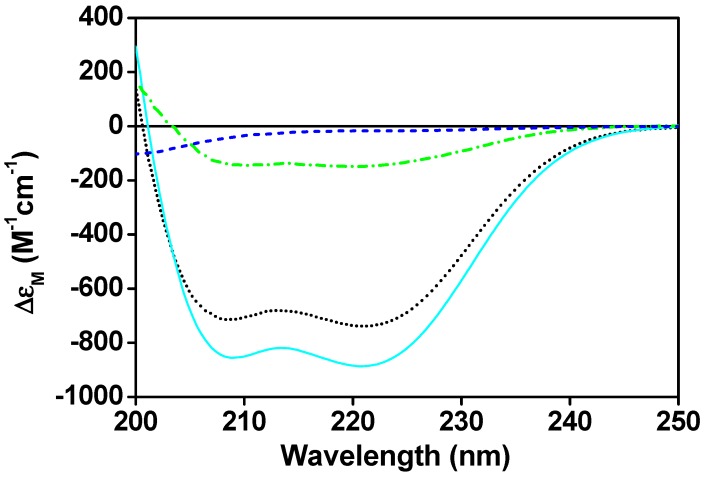
The secondary structure of CaM/CaNp complex. Far-UV CD spectra of CaM (black dotted line), free CaNp (blue dashed line), the complex of CaM and CaNp in a 1:1 molar ratio (cyan solid line), and the difference between the CaM/CaNp complex and free CaM (green dashed/dotted line). The CD spectra were taken at the concentration of 10 µM CaM and/or 10 µM CaNp in 100 mM KCl, and 2 mM CaCl_2_ at pH 6.5.

The change in the secondary structure of CaM upon the complex formation with CaNp has been investigated by CD spectroscopy (Figure 1). The free CaM displayed a typical helical structure with minima molar CD absorption coefficient (Δε_M_) at 222 nm and 208 nm, whereas the CaNp alone showed mostly random-coil structure in the aqueous solution.

When an equivalent concentration of CaNp was added to the CaM solution, the negative molar ellipticity at 208 nm and 222 nm increased. When more than one equivalent concentration of CaNp was added to the CaM solution, the negative molar ellipticity at 208 nm and 222 nm remained almost the same. The data suggested the complex formation with the molar ratio 1:1. In addition, the results showed an increase of α-helical content upon complex formation. Structural information of the CaM-peptide complexes extracted from the protein data bank (PDB) indicated that CaM itself usually did not gain α-helical structure upon peptide binding. Therefore, we assumed that the increase in α-helical content was mostly attributed by the bound CaNp. The spectral difference between the CaM/CaNp complex and free CaM displayed in Figure 1 was used to estimate the α-helical content in the CaNp when associated with CaM. An approximately 71% α-helical content in the bound CaNp was estimated by the Dichroweb program [64,65], corresponds to a 17-residue α-helix in CaNp. This observation was in good agreement with many CaM-binding peptides in the 1:1 binding class.

### 2.2. Hydrodynamic Properties of CaM/CaNp Complex by Dynamic Light Scattering (DLS) and NMR Spectral Line Width

**Figure 2:**
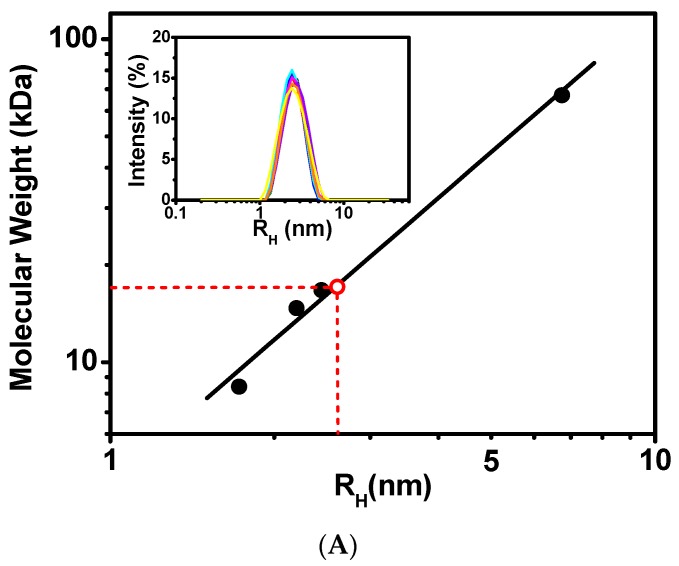

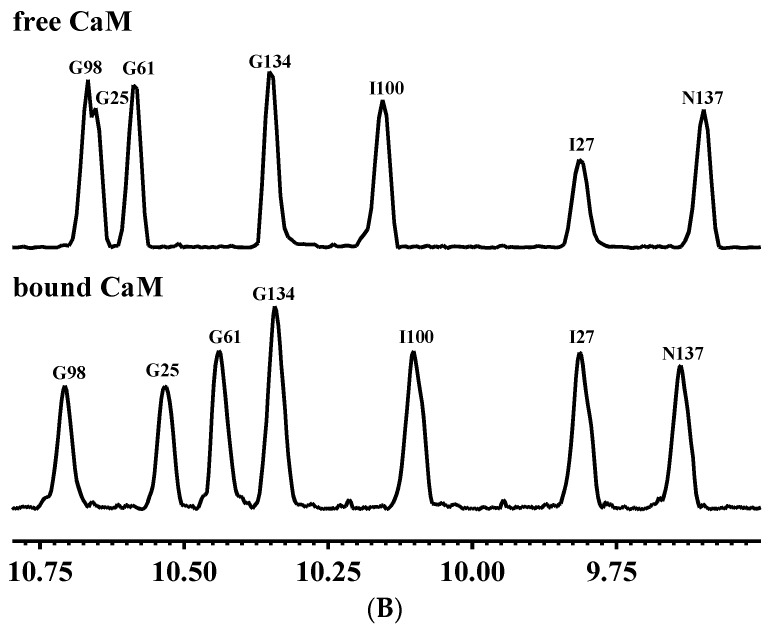
Stoichiometric ratio measurements of CaM/CaNp complex by DLS and NMR. (**A**) Dynamic Light Scattering (DLS) measurements on standard proteins and CaM/CaNp complex. A double logarithmic calibration curve of R_H_ versus M_r_, shown in black line was generated using DLS measurements of standard proteins including BSA (R_H_ 6.75 nm, M_r_ 67 kDa), CaM (R_H_ 2.44 nm, M_r_ 16.7 kDa), lysozyme (R_H_ 2.20 nm, M_r_ 14.7 kDa), and ubiquitin (R_H_ 1.7 nm, M_r_ 8.4 kDa), which were shown in black circles. Hydrodynamic radius (R_H_) distribution of CaM/CaNp complex was determined by DLS. Nine DLS measurements were performed for CaM/CaNp complex, and the average R_H_ for CaM/CaNp complex is 2.61 nm (inset). The M_r_ of CaM/CaNp was then estimated to 17.2 kDa by linear intrapolation by red dash lines. (**B**) 1D NMR spectral linewidth measurements on free CaM and CaM/CaNp complex. The average half-width of NH resonance of G61, G134, I100 I27, and N137 for the bound CaM is 15.5 Hz; the average half-width of those NH resonances for the free CaM is 16.0 Hz.

The hydrodynamic properties of CaM/CaNp complex were investigated by dynamic light scattering (DLS) and NMR spectroscopy. The inset in Figure 2A presents the time-average distribution of hydrodynamic radius (R_H_) of CaM/CaNp complex obtained from nine DLS measurements. The data showed that the complex formed a single species with average R_H_ 2.61 nm in solution with no evidence of polymeric forms with larger R_H_ values. Calibrated with molecular weight standards shown in Figure 2A the R_H_ of CaM/CaNp complex corresponded to a globular protein of M_r_ 17.2 kDa, roughly consistent with the theoretical M_r_ calculated from the sequence of CaM and CaNp in a 1:1 ratio (19.5 kDa).

In addition to the DLS technique, linewidths in the NMR spectrum are also frequently used to probe the effective hydrodynamic properties of biomolecules. The larger molecular size causes an increase in the effective hydrodynamic volume, leading to a larger rotational correlation time (τ_c_) and a corresponding increase in the NMR spectral linewidth. As shown in Figure 2B, the NMR resonances of CaM when associated with CaNp were sufficiently sharp compared to those in free CaM, the half-width of NH resonance of G61, G134, I100 I27, N137 of bound CaM was similar to or even smaller than the half-width of resonance in free CaM respectively. Similar phenomenon were found in 2D ^1^H-^15^N HSQC spectra, the average half-width of 39 isolated NH resonances of bound CaM was similar to the average half-width of resonances in free CaM (Table S2). Our data from the DLS and NMR line width experiments indicated that CaM/CaNp complex existed in a 1:1 ratio form in solution. The domain-swapping dimeric CaM/CaNp complex found in the crystal structures (PDB 2R28 and 2W73) with 2:2 ratio was not observed in solution.

### 2.3. Activity of CaN is Activated by CaM

**Figure 3:**
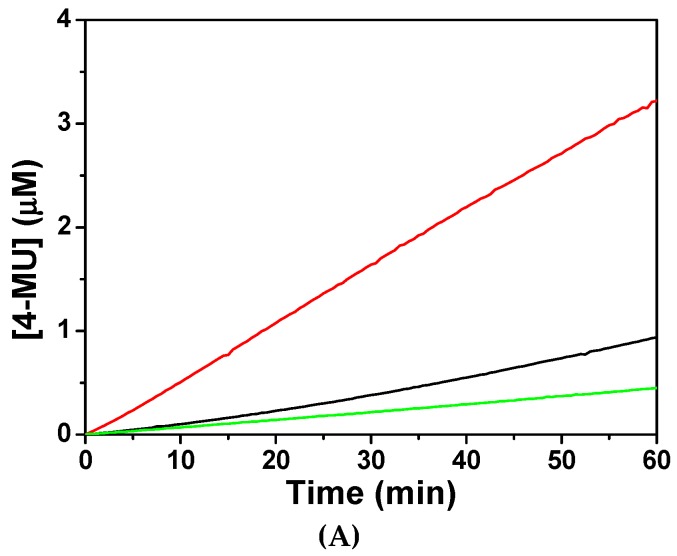

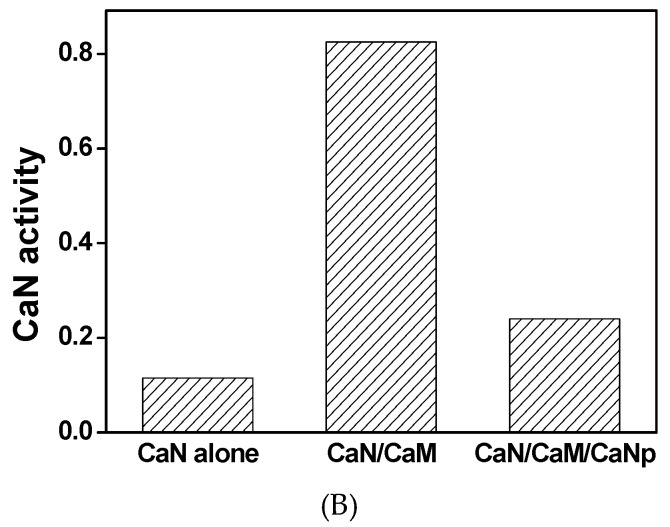
The phosphatase activity of CaN activated by the presence of recombinant CaM. (**A**) The hydrolysis of a fluorogenic substrate, 4-methylumbelliferyl-phosphate (4-MUP) by CaN was monitored spectrofluorometrically with excitation and emission wavelengths at 365 nm and 445 nm, respectively. The fluorescence intensity was converted to the concentration of the product, 4-MU using a calibration curve as described in Materials and Methods. The hydrolysis time curve of 4-MUP assayed by CaN alone, CaN with equimolar CaM, and CaN with equimolar CaM and CaNp were shown in green, red, and black respectively; (**B**) The average specific activity of CaN against 4-MUP in the absence or presence of CaM and CaNp. The averaged specific phosphatase activity of CaN was calculated over the time period of 60 min, as µmol 4-MU produced per hour per µg CaN.

In order to understand the mechanism of CaM to activate CaN phosphatase, the phosphatase activities of CaN were assayed fluorometrically. As shown in Figure 3, when assayed without CaM, CaN was much less active with average activity 0.12 µM/h product produced per µg CaN used. The low residual activity of CaN was probably due to the existence of minor proteolytic form of CaN A subunit which lacks the regulatory domain.

The specific activity of CaN increased to 0.83 µM/h when equivalent concentration of CaM was added to the reaction. However, the specific activity of CaN was 0.24 µM/h when CaM was pretreated with equivalent concentration of CaNp. Apparently, CaM loses most of its ability to activate CaN in the presence of CaNp. Our data indicated that both CaN and CaNp competed for the binding to CaM, however, the binding of CaNp is much stronger than CaN to CaM.

### 2.4. Resonance Assignments of the Complex and Chemical Shift Deviation of CaM upon Binding to CaNp

**Figure 4:**
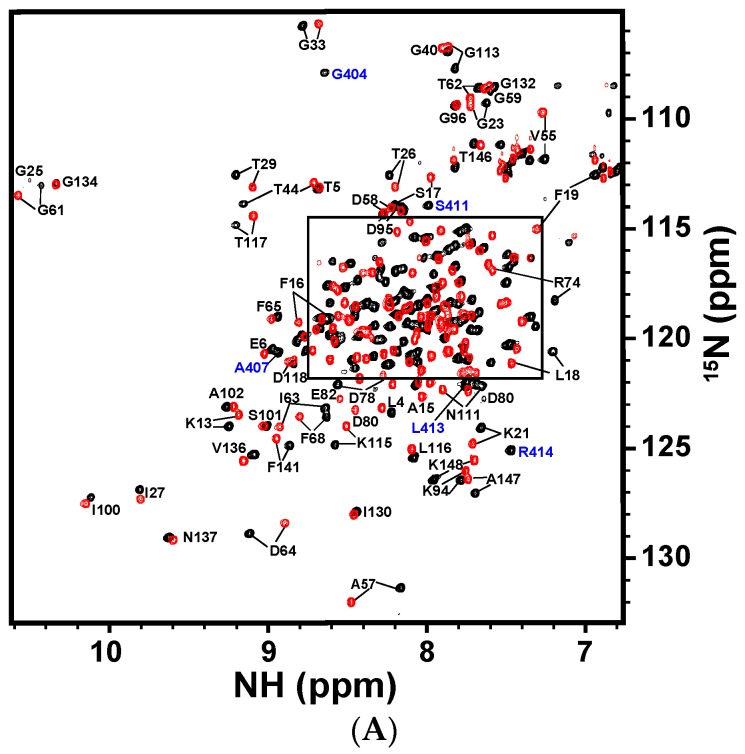

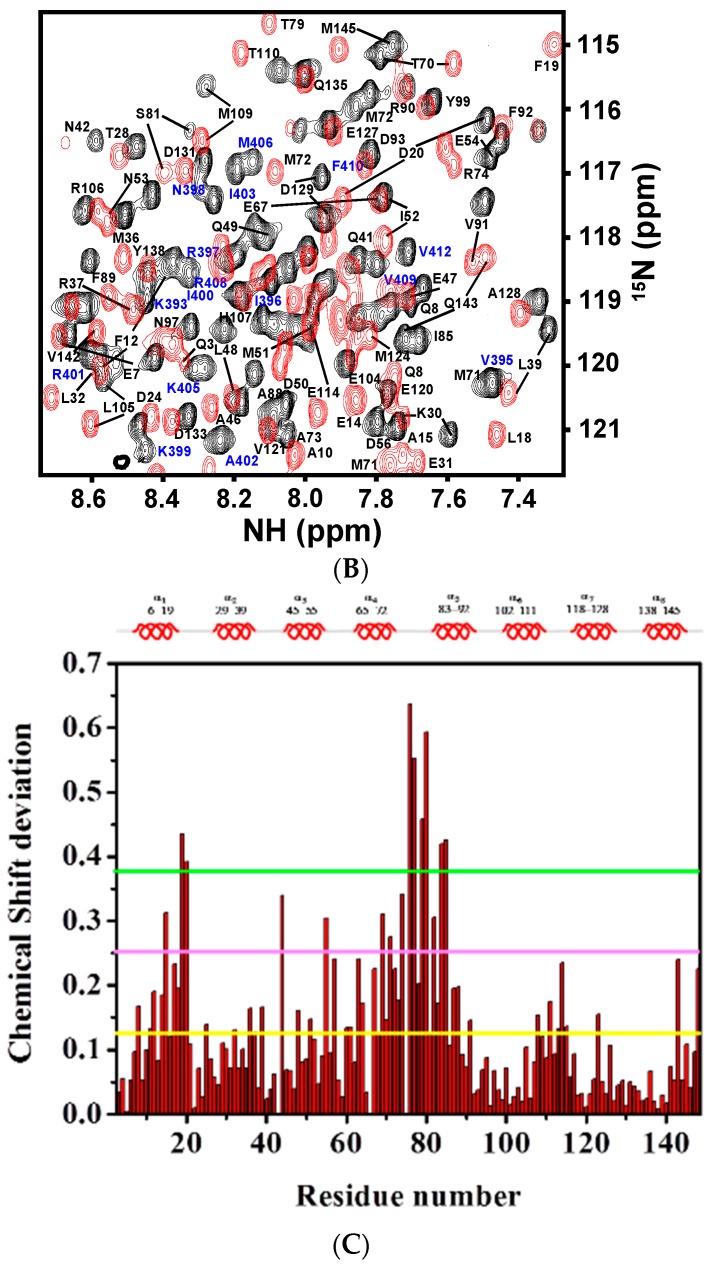
Two-dimensional ^1^H-^15^N HSQC spectrum of CaM in complex with CaNp and chemical shift deviation. (**A**) The superimposed full ^1^H-^15^N HSQC spectra of free CaM and CaM/CaNp complex. The spectra were acquired at 310 K in 20 mM KCl, at pH 6.5. The ^1^H-^15^N crosspeaks of free CaM and CaM/CaNp were shown in red, and in black, respectively. Assignments of the ^1^H-^15^N crosspeaks of CaM and CaNp are labeled in black and blue, respectively. The box region in the full spectrum was expanded and shown in (**B**) for the purpose of clarity; (**C**) The overall chemical shift perturbation, Δδ_residue_ of CaM upon association with CaNp was plotted against residue number. Δδ_residue_ of each residue was calculated as described in the Materials and Methods. The average of Δδ_residue_ for all residues of CaM upon association with papain was calculated and shown as the yellow horizontal solid line (0.126 ppm). The magenta and green horizontal solid lines represented two- and three-fold averaged Δδ_residue_, respectively. The most deviated regions with chemical shift deviation larger than three-fold of average Δδ_residue_ are located in helix 1 and central tether regions.

The backbone ^1^H-, ^13^C-, and ^15^N-resonances of CaM and CaNp in the complex were assigned respectively from an array of triple resonance NMR experiments listed in the Materials and Methods section. All of the ^1^H-, ^13^C- and ^15^N-backbone resonances of the complex were assigned, except N-terminal residues. The aliphatic side-chain proton and carbon chemical shifts were assigned from C(CO)NH, H(CCO)NH, HCCH-TOCSY, and HCCH-COSY spectra. About 95% of the proton and carbon resonances were assigned. The full resonance assignments were deposited in the BioMagBank with accession number 15624. Figure 4A,B show the 2D ^1^H-^15^N HSQC spectrum of CaM/CaNp complex and the resonances are labeled.

Since chemical shifts are sensitive to changes in chemical environments caused either by proximity to the interaction surface or by structural readjustments, they are frequently used as atomic resolution probes to monitor the interaction between biomolecules. We herein used chemical shift perturbation to map the regions with structural adjustments of CaM when associated with CaNp. The normalized chemical shift deviation of each residue, Δδ_residue_ was calculated as described in Materials and Methods and listed in Table S1. The normalized chemical shift deviation of CaM upon association with CaNp, Δδ_residue_ was plotted against residue number as shown in Figure 4C. The average Δδ_residue_ for all residues of CaM upon association with CaNp was 0.126 ppm. Although the chemical shifts deviated in both N- and C-domains of CaM, but more in the N-domain. The most perturbed residues of CaM with Δδ_residue_ larger than three folds of the averaged Δδ_residue_ were found in two regions, residue 19–20 (in helix 1) and residue 76 to 85 (in the inter-domain region). Our data suggested that these two regions in CaM undergo structural rearrangements upon association with CaNp.

### 2.5. Solution Structure of the Complex of CaM/CaNp and Its Novel Features

The solution structures of CaM/CaNp complex were calculated using CYANA 2.1. There were 2491 NOE distance restraints used in the calculation, including 1990 and 373 restraints for CaM and CaNp, respectively, and 128 intermolecular distance restraints between CaM and CaNp. In addition to NOE distance restraints, 72 distance restraints for hydrogen bonds, 22 distance restraints between the Ca^2+^ ions and the side chains of Asp and Asn residues of CaM, and 294 dihedral angle restraints were used for final calculation of the structures. An ensemble of 20 conformers with lowest restraint violations was selected for further analysis. The quality of the structural ensemble was evaluated by the program PROCHECK-NMR, in which 98.2% of the residues were in the most favored and additionally allowed regions, only 1.8% in generously allowed region, and no residues in disallowed region in the Ramachandran plot. Table 1 summarizes the input data and structure statistics.

**Figure 5:**
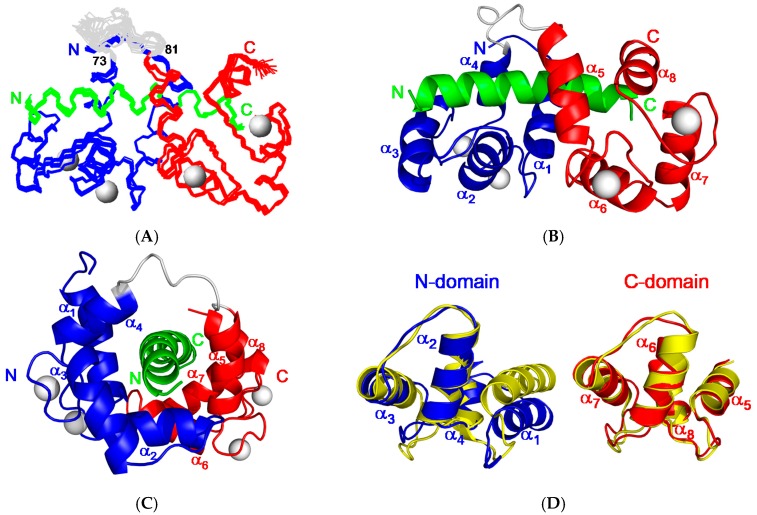
Solution structure of CaM in complex with CaNp. (**A**) The ensemble of 20 best structures of the complex of CaM and CaNp is superimposed. The backbone structures of the N-domain, C-domain, and tether region of CaM are colored coded in blue, red, and grey respectively. Ca^2+^ ions are shown as grey spheres. The CaNp is colored coded in green. The N- and C-termini of CaM and CaNp are labeled as N and C respectively; (**B**) The ribbon representation of CaM/CaNp complex structure. The complex structure shows an ellipsoidal shape with 8 α-helices (α_1_–α_8_) of CaM wrapping the CaNp helix; (**C**) Side view of the ribbon representation of CaM/CaNp complex structure. CaNp shows a curved helical conformation centered at the residue Gly^404^; (**D**) The structural changes of the two domains of CaM upon binding to CaNp. The structure of free CaM (in yellow) superimposed with the structure of CaM when associated with CaNp. The N-domain and C-domain of CaM in the bound form is colored in blue and red respectively. N-domain of CaM, especially helix I (α_1_) undergoes larger structural changes upon association with CaNp, whereas C-domain of CaM remains unchanged.

The final refined ensembles of 20 lowest energy structures have been deposited into the protein data bank (PDB) with accession code 2JZI. The best-fit superposition of the 20 best backbone structures of CaM/CaNp complex and black respectively is shown in Figure 5A, the ensemble of 20 structures has root mean square deviations (RMSD) of 0.66 Å for backbone and 1.14 Å for all atoms (residue 5 to 145 for CaM and residue 392 to 413 for CaNp). The amino (residue 1 to 4), and carboxyl (residue 146 to 148) termini of CaM, the linker region connecting the two homologous domains of CaM (residue 73–81) are not well-defined. Two ribbon diagrams of the averaged structure in different orientations are shown in Figure 5B,C. The complex structure shows an ellipsoidal shape with eight α helices (α_1_–α_8_) of CaM wrapping around the CaNp helix.

**Figure 6:**
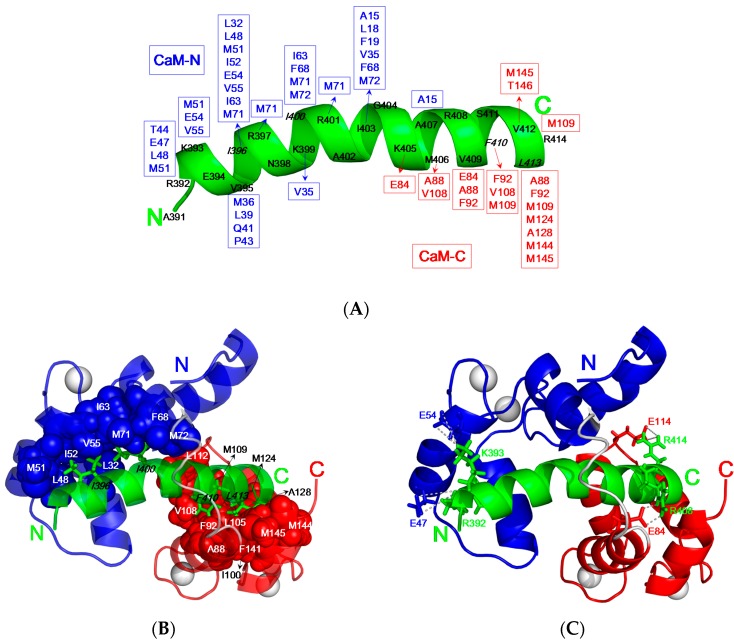
Intermolecular interactions between CaM and CaNp. (**A**) Summary of the observed NOEs between CaM and CaNp. CaNp is shown as a green helical ribbon. Interacting residues in N-domain and C-domain of CaM are labeled in blue and red, respectively; (**B**) The hydrophobic interactions between CaM and CaNp. Two intermolecular hydrophobic clusters were found in the complex of CaM/CaNp. Bulky side chains of Ile^396^and Ile^400^ of CaNp (shown in green sticks) anchored in the hydrophobic pocket of N-domain of CaM (shown in blue spheres). Bulky side chains of Phe^410^, and Leu^413^ of CaNp (shown in green stick) anchored in the hydrophobic pocket of C-domain of CaM (shown in red spheres); (**C**) The potential electrostatic interactions between the negatively charged residues of CaM (Glu^47^, Glu^54^, Glu^84^, and Glu^114^) and the positively charged residues of CaNp (Arg^392^, Lys^393^, Arg^408^, and Arg^414^) are shown.

As compared to the backbone of free CaM [9], the C-terminal domain of CaM (residue 84–145, C-domain) remains mostly unchanged with the RMSD value 1.0 Å upon association with CaNp as shown in Figure 5D. However, the N-terminal domain (residue 5–72, N-domain) exhibits a much larger conformational change upon association with CaNp, with the RMSD value 2.3 Å, helix I (α_1_) is pushed away from helices II to IV (α_2_ to α_4_). Besides the displacement of α_1_, the inter-domain linker region, from residue 73 to 81 of CaM unwinds to a flexible loop. The large structural change in the regions of α_1_ and inter-domain linker of CaM is in accord with the chemical shift deviation data. Upon associated with CaM, CaNp folds up to a α-helical structure from random coil in the region of residue 392 to 413. Unlike the most determined CaM/peptide structures, the structure of bound CaNp consists of two helical segments, N-helix and C-helix, separated by the tether residue Gly^404^ with a 17° inter-helical angle. The curvature in center of the helical CaNp may reduce the stretching force between the two domains of CaM to accommodate the longest binding helix in CaM target sequences.Extensive intermolecular NOEs between side chains of CaNp and CaM were observed from the isotope-separated NOESY-HSQC spectrum and summarized in Figure 6A. The spacing and position of bulky hydrophobic residues of the target sequence are important features in characterizing the binding modes of CaM and target sequences. The most abundant NOE pairs were found from two major hydrophobic residues, Ile^396^, and Leu^413^ and two minor hydrophobic residues, Ile^400^, and Phe^410^ which exhibit a novel 1-5-15-18 binding type. The intermolecular NOE pairs were found between the residues in the N-helix of CaNp and the N-domain of CaM, and between C-helix of CaNp and the C-domain of CaM. Apparently, the binding orientation of CaNp with respect to the two globular domains of CaM is opposite to those structures in the prototypical 1–14 and in 1–10 binding types. Instead, the binding orientation of CaNp with respect to CaM is similar to the CaMKK peptide in the 1–16 binding mode.

As shown in Figure 6B, the hydrophobic surface of N-domain of CaM was occupied by the side chains of Ile^396^ and Ile^400^ of CaNp which located in the N-helix of CaNp. These two anchor residues of CaNp form hydrophobic contacts with numerous hydrophobic residues (Leu^32^, Leu^48^, Ile^52^, Val^55^, Ile^63^, and Phe^68^) and three methionine residues (Met^51^, Met^71^, and Met^72^) in the N-domain of CaM. On the other hand, the hydrophobic surface of C-domain of CaM was in close contacts with the side chains of Phe^410^, and Leu^413^ of CaNp which are located in the C-helix of CaNp.

Interestingly, mediator residue Ile^403^ located in the middle of CaNp is in close contacts with the hydrophobic side chains from both domains of CaM including Ala^15^, Leu^18^, Phe^19^, Val^35^, Phe^68^, and Met^72^ from N-domain and Leu^112^ from C-domains of CaM. The intermolecular hydrophobic interactions between Ile^403^ of CaNp and Ala^15^, Leu^18^, Phe^19^, located in the helix I of CaM explain the large displacement of α_1_ respective to the rest of N-domain when associated with CaNp and in accord with the chemical shift deviation data. In addition to the hydrophobic interactions, the electrostatic complementarity between CaM and CaNp also plays an important role in the binding affinity and the orientation of CaNp to CaM. The intermolecular electrostatic interactions involve the positively charged Arg/Lys residues of CaNp and the negatively charged Glu residues of CaM. Four intermolecular electrostatic pairs, including Arg^392^/Glu^47^, Lys^393^/Glu^54^, Arg^408^/Glu^84^, and Arg^414^/Glu^114^ between CaNp and CaM were found to stabilize the complex structure (Figure 6C).

### 2.6. Comparison of the Solution Structure of CaM/CaNp to Other CaM/Target Peptide Structures

**Figure 7:**
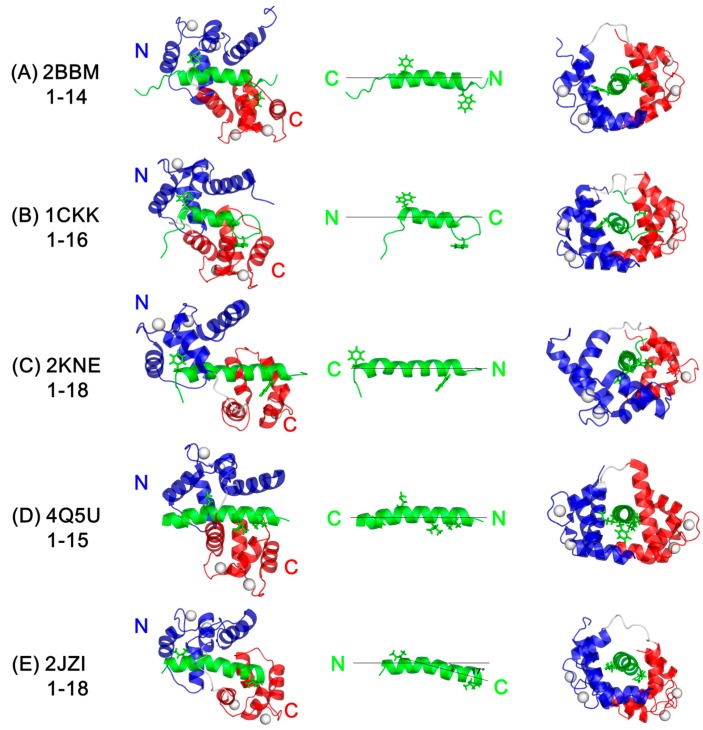
Comparison of relative orientation of N- and C-domain of CaM upon binding to different target peptides. (**A**) CaM and the target sequence of skeletal muscle myosin light chain kinase (PDB 2BBM); (**B**) CaM and the target sequence of CaM-dependent kinase kinase (PDB 1CKK); (**C**) CaM and the target sequence of plasma membrane calcium pump (PDB 2KNE); (**D**) CaM and CaNp in crystal form (PDB 4Q5U); (**E**) CaM and CaNp in solution form (PDB 2JZI). In order to compare the relative position of the C-domain of CaM in the structure of 2JZI, 2BBM, 1CKK, 2KNE, and 4Q5U, the position of the N-domain of CaM in these structures was fixed.

In order to show the novel features of CaM/CaNp complex, the representative structures of CaM/target peptide complexes in different CaM-recognition modes including 1–14, 1–16, and 1–18 binding types were compared to the solution structure of CaM/CaNp. The ribbon diagrams of prototypical structures of CaM associated with the target peptide from myosin light chain kinase (PDB ID: 2BBM), CaM-dependent kinase kinase (PDB ID: 1CKK), plasma membrane calcium pump (PDB ID: 2KNE), and calcineurin (PDB ID: 4Q5U in crystal form; 2JZI in solution form) are shown in Figure 7. The backbone conformation of each domain of CaM in 2JZI resembles to free CaM and other CaM/target peptide structures including 2BBM, 1CKK, 2KNE, and 4Q5U. The backbone RMSD value is in the range of 1.0–1.8 Å for C-domain (residue 82–145), and 2.1–2.5 Å for N-domain (residue 5–72), respectively. The larger RMSD in N-domain of CaM is caused by the displacement of helix I upon binding to CaNp. If the coordinates of helix I are excluded, the RMSD for N-domain (residue 28–72) is greatly reduced to 1.2–1.8 Å. Other novel features of the solution structure of CaM/CaNp complex include the conformation of bound CaNp, and the relative position of the two globular domains. Different from the target peptides in the previous determined CaM/peptide complex structures, bound CaNp in 2JZI forms a novel V-shaped helical structure with a kink at the residue Gly^403^ as shown in Figure 7E. The relative position of the two homologous domains of CaM in CaM/target peptide complexes is in a ‘C2-like’ symmetry except the solution structure of CaM/CaNp complex. The bend in the bound CaNp helix causes a certain deviation of the relative orientation of the two homologous CaM domains from C2-like symmetry. In order to characterize the relative position of the two homologous domains in different CaM/target peptide complexes, a screw rotation axis passing through the α-helical axis of the target peptide is chosen (Figure 7). To superimpose the N-domain onto C-domain of CaM along this screw axis, a roughly C2 symmetry is found in the structures of 2BBM, 1CKK, 2KNE, and 4Q5U. However, in the case of CaM/CaNp structure in solution form, an additional 17° rotation angle had to be applied.

There are differences between the structures of CaM/CaNp in solution form (2JZI) and in crystal form (4Q5U). The crystal structure of CaM/CaNp in 4Q5U can be classified as 1–15 CaM-target binding mode according to the spacing between the two major hydrophobic anchors in the target sequence; however, the solution structure of 2JZI shows 1–18 CaM-target binding mode. The relative orientation of the helical target sequence respective to CaM is also different. In the crystal structure of 4Q5U, the hydrophobic anchors in the N-terminal and C-terminal of target sequence were engulfed in the hydrophobic pocket of the C- and N-terminal domain of CaM, respectively. On the contrary, the orientation of the helical target peptide with respect to the two domains of CaM in solution is opposite to that in crystal. In addition, the ionic interactions between the target peptide and CaM are different. In the crystal structure 4Q5U, four intermolecular electrostatic pairs, including Arg392/Glu114, Lys393/Glu127, Arg408/Glu84, and Arg414/Glu47 between CaNp and CaM were found. In the solution structure of 2JZI, four intermolecular electrostatic pairs, including Arg392/Glu47, Lys393/Glu54, Arg408/Glu84, and Arg414/Glu114 were found.

### 2.7. The Activation Processes of CaN by CaM

**Figure 8:**
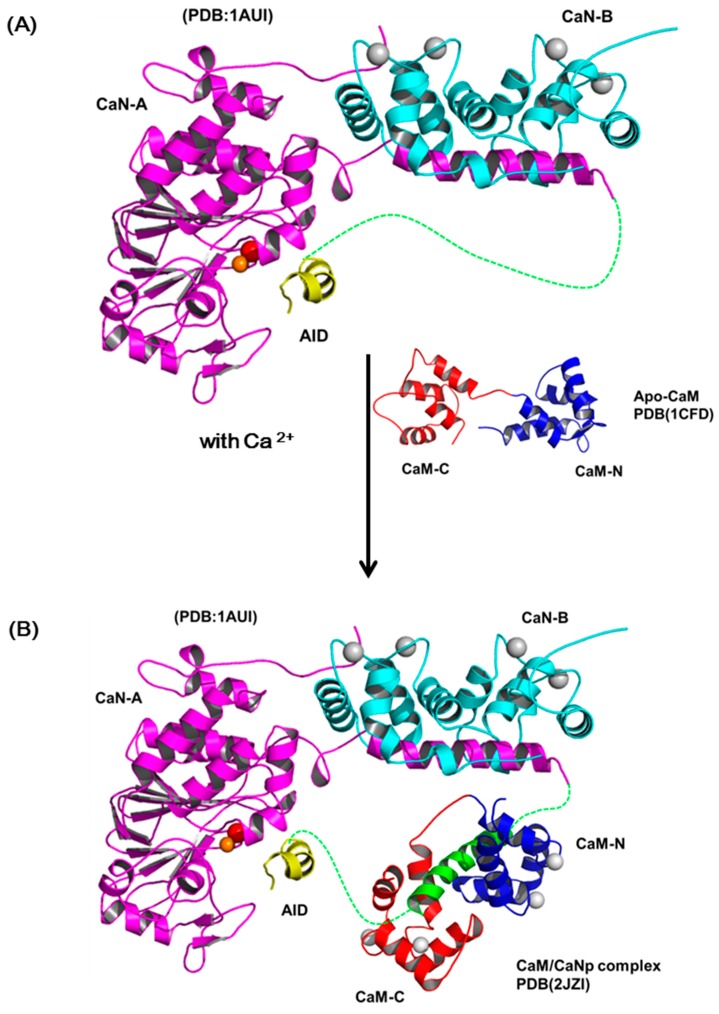

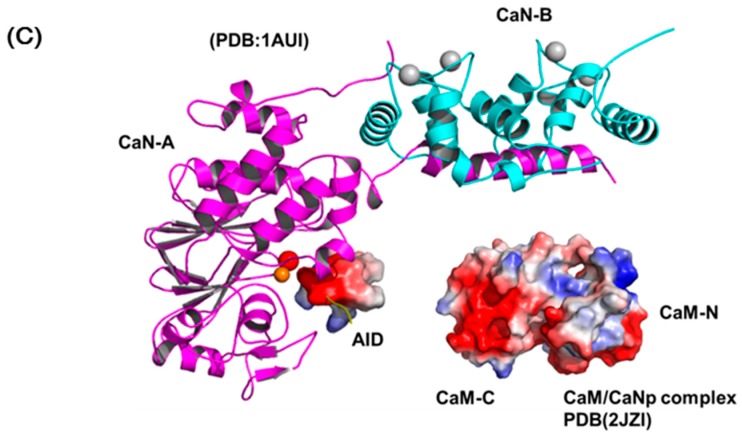
The schematic diagram of the activation processes of CaN. (**A**) Ribbon representation of the structure of human CaN (1AUI). The CaN-B is colored coded in cyan. The catalytic domain, AID of CaN-A is colored coded in magenta and yellow. The region connecting the BBD to AID is invisible in the electron density map (draw in green dashed line). (**B**) The complex formation of CaM and the target sequence of CaN. (**C**) The charge distribution of CaM/CaNp complex and AID. Positive and negative electrostatic charge is colored in blue and red, respectively. Both surfaces are mostly negatively-charged, especially in the C-domain of CaM/CaNp complex. The electrostatic repulsion between CaM/CaNp complex and AID may result in the displacement of AID from active site to full activation.

The most accepted model for the activation of CaN by Ca^2+^ and CaM includes several sequential changes in conformations [49,66]. In summary, the conformational change is triggered by occupancy of calcium ions to the low affinity sites of CaN-B subunit which causes the dissociation of the CaM target domain from the CaN-B binding domain. Subsequently, CaM recognizes this target region and leads to the displacement of auto-inhibitory domain (AID) from the active site and full activation. Although this activation model is in accord with most biochemical and biophysical results, there is limited structural information reported to prove the details of the structural changes in the activation process. Here, we reported the solution structure of CaM/CaNp to put back the missing pieces for the activation action of CaM on CaN. Figure 8 shows a schematic diagram for the activation of CaN combining previous models and our data reported here. The regulatory domain of CaN-A subunit is mostly unstructured and invisible in the crystal structures in the region ranged from residue 369 to 521, only a short AID helix located near the active site was found (PDB ID: 1AUI) (Figure 8A). The hydrophobic surface of CaM recognizes the hydrophobic anchors in the target sequence in CaN, and further transforms the target sequence to mostly helical structure. Based on our structural and chemical shift deviation data (Figure 4C and Figure 5D), we found much less structural/chemical changes in the C-domain of CaM upon association with the target sequence of CaN.

Since the two domains of CaM exhibit subtle differences in Ca^2+^ affinity, in the presence of limited concentration of calcium, here only the high affinity Ca^2+^-binding sites in C-domain of CaM are saturated. We therefore propose that the hydrophobic interactions between the Ca^2+^-saturated C-domain of CaM and the C-terminal half of the target sequence of CaN, which contains more hydrophobic residues provide the driving force for the initial recognition. Subsequently, the target sequence of CaN undergoes a conformational transition from unstructured to helical and further improves its structural complementarity and binding affinity to CaM. Meanwhile, upon binding with the target sequence, CaM also undergoes conformational adjustment and the affinity to Ca^2+^ greatly increases, leading to the final structure of the full Ca^2+^-saturated CaM/CaNp complex in an ellipsoidal shape (Figure 8B). Finally, the charge distribution on surfaces of CaM/CaNp complex and AID is mostly negatively charged, especially on the surface of the C-domain of CaM/CaNp complex. The electrostatic repulsion between CaM/CaNp complex and AID may result in the displacement of AID from active site to full activation (Figure 8C).

## 3. Materials and Methods

### 3.1. Cloning, Expression and Purification of CaM and CaNp 

The cDNA of chicken CaM was subcloned into a modified pET29c expression vector. The construct was verified by DNA sequencing, and then transformed into *E. coli* BL21 (DE3) host and expressed. The recombinant CaM was purified by a hydrophobic, phenyl sepharose column. ^13^C, ^15^N-labeled CaM was expressed in a modified M9 medium with ^15^NH_4_Cl and ^13^C-glucose as sole ^15^N and ^13^C sources. The gene coding for the CaM-CaNp hybrid molecule was synthesized by a series of PCR reactions. The 5′-end primer contained an *Nde*I restriction enzyme cleavage site, and the 3′-end primers contained the sequences coded CaNp followed by stop condon and an *Xho*I restriction enzyme cleavage site. The PCR-amplified CaM-CaNp hybrid gene was then subcloned into a modified pET29c expression vector. The construct was verified by DNA sequencing of the entire coding region and the cloning sites. The recombinant CaM-CaNp hybrid molecule comprises the sequences of CaM, CaM-binding domain of CaN (CaNp, ^391^ARKEVIRNKIRAIGKMARVFSVLR^414^), and a factor Xa cleavage site (IEGR) in between. The plasmid coding for CaM-CaNp hybrid molecule was transformed into *E. coli* strain BL21 (DE3). The transformed cells were grown at 37 °C to 1 OD_600nm_ in LB medium, 0.4 mM IPTG was then added to induce gene expression. The harvest cells were resuspended in 25 mM Tris-HCl buffer pH 8.0, 1 mM EDTA, and 0.1 mM PMSF, and disrupted by sonication. After the cell debris had been pelleted by centrifugation, the whole cell lysate containing CaM-CaNp hybrid molecules were separated by a Q-Sepharose ion exchange column, followed by a size exclusive column (Hiload 16/60 Superdex 75, GE Healthcare, Wauwatosa, WI, USA). The purified CaM-CaNp hybrid molecules ran as a single band in SDS-PAGE. The linker (IEGR) in between the sequences of CaM and CaNp was then cleaved by factor Xa according to the instructions of manufacturer (GE Healthcare). CaNp was separated from CaM by ultrafiltration with an YM-10 membrane (Millipore-Sigma, St. Louis, MO, USA) at pH 2. The purified CaNp (>95%) was confirmed by analytical HPLC and mass spectrometry. The ^13^C-^15^N-labeled CaM-CaNp hybrid molecules were expressed in a modified M9 medium with ^13^C-glucose and ^15^NH_4_Cl as sole ^13^C and ^15^N sources (Cambridge Isotope Inc., Tewksbury, MA, USA). The ^13^C-^15^N-labeled CaNp was obtained using the same separation procedures as non-labeled CaNp.

### 3.2. Purification of CaN Enzyme from Bovine Brain

CaN enzyme was isolated from bovine brain cerebra as previously described with modifications [67]. Bovine brain cerebra were obtained fresh from a local abattoir and stored at −20 °C until needed. The CaN enzyme was purified by three steps including Affi-gel blue (BioRad, Hercules, CA, USA), CaM-sepharose affinity, and mono-Q anion exchange (GE Healthcare) chromatography. Bovine brain extract was loaded onto Affi-gel blue, and eluted with buffer A (50 mM Tris-HCl, 3 mM MgSO_4_, 0.5 mM DTT, 1 mM EDTA, and 0.02% NaN_3_ at pH 7.8). Fractions containing CaN enzyme were pooled and loaded onto a CaM-Sepharose affinity column which had equilibrated with buffer B (50 mM Tris-HCl, 3 mM MgSO_4_, 0.5 mM DTT, 0.1 mM CaCl_2_, and 0.02% NaN_3_ at pH 7.8). The affinity column was extensively washed with buffer B supplemented with 0.5 M NaCl and then eluted with buffer A. The pooled fractions from the affinity column were then loaded onto a mono-Q column, eluted with a linear NaCl gradient. The purity of CaN enzyme was higher than 80% by SDS-PAGE with two major bands, 60 kDa (CaN A subunit) and 20 kDa (CaN B subunit). A minor band, the partial proteolytic fragment of CaN A subunit with 48 kDa was also observed. The purified CaN enzyme was stored with 50% glycerol at -20°C.

### 3.3. Activity Assay of CaN

Phosphatase activities of CaN were assayed fluorometrically using 4-methylumbelliferyl-phosphate (4-MUP, Sigma, St. Louis, MO, USA) as the substrate. The basal activity of CaN was determined using 200 µM substrate and 25 nM enzyme CaN in the buffer containing 50 mM Tris pH 8.6, 0.5 mM DTT, 1 mM MgCl_2_, and 0.3 mM CaCl_2_ at 25 °C. The CaM-dependent activation of the activity of CaN was measured by the addition of 25 nM CaM to the same reaction conditions described above. Fluorescence data were recorded using an Aminco Bowman (ABII) spectrofluorometer (Thermo Spectronic Inc., Rochester, NY, USA). The formation of the product, 4-MU, was monitored with excitation wavelength at 365 nm, and emission wavelength at 445 nm. Data were collected over a time course of 75 mins. A calibration curve was generated by measuring the fluorescence intensities of solutions containing various concentrations of 4-MU under the same assay conditions. The fluorescence intensities of the test samples were converted to the 4-MU concentration according to the calibration curve. The phosphatase activity of CaN is defined as µmol 4-MU produced per hour per µg CaN used.

### 3.4. NMR Sample Preparation

The NMR sample was prepared in the following procedures, 10 mg CaM was dissolved into 1 mL solution containing 90% H_2_O/10% D_2_O, 10 mM CaCl_2_, 20 mM KCl, 0.02% NaN_3_, at pH 6.5. The appropriate amount of CaNp stock solution (2 mM) was added gradually into the CaM solution with gentle mixing to ensure the CaM/CaNp complex formation. The solution was then concentrated by Centricon-10 ultrafiltration apparatus (Millipore Inc.) to a final concentration of CaM/CaNp complex about 1.1 mM. The excess CaNp molecules were also removed by this procedure. The final CaM/CaNp complex sample solution was transferred to 5 mm Shigemi NMR tubes (Shigemi Co., Tokyo, Japan) for recording NMR spectra.

### 3.5. NMR Spectroscopy

The NMR experiments were performed at 310 K on an Avance II 600 MHz spectrometer (Bruker BioSpin, Rheinstetten, Germany) at National Dong Hwa University, or a 800 MHz spectrometer (Bruker BioSpin, Rheinstetten, Germany) at the High-Field Biomacromolecular NMR Core Facility, Academia Sinica. All spectra were processed by TOPSPIN on Linux workstations. All the chemical shifts were referenced to internal DSS in ^1^H dimension, and calibrated using the gyromagnetic ratio 0.101329118 (^15^N/^1^H) in ^15^N dimension and 0.251449530 (^13^C/^1^H) in ^13^C dimension [68]. Backbone and side chain assignments of CaM and CaNp in the complex form were assigned separately by the NMR spectra obtained from ^13^C-^15^N-labeled CaM associated with non-labeled CaNp and non-labeled CaM associated with ^13^C^15^N-labeled CaNp samples. Backbone and side chain sequential assignments were obtained using the following standard experiments: 2D ^15^N-^1^H-HSQC, 2D ^13^C-^1^H-HSQC, 3D HNCO, 3D HN(CA)CO, 3D HNCA, 3D CBCANH, 3D CBCA(CO)NH, 3D CC(CO)NH, 3D HBHA(CBCACO)NH, 3D HCC(CO)NH, 3D HCCH-COSY and 3D HCCH-TOCSY. The assignments of aromatic side chains in CaM/CaNp complex were obtained by homonuclear 2D spectra including DQF-COSY, TOCSY, and 3D ^15^N-separated NOESY-HSQC spectra. The side chain amide groups of Asn and Gln were assigned on the basis of NOEs between ^1^H_γ_ or ^1^H_δ_ and ^1^H_β_ observed in the 3D ^15^N-separated NOESY-HSQC spectrum. The NOE distance restraints were obtained from 3D ^15^N-separated and ^13^C-separated NOESY-HSQC spectra of the ^13^C^15^N-labeled CaM associated with ^13^C^15^N-labeled CaNp sample.

### 3.6. Structure Calculation and Analysis

NOE distance restraints were derived from 3-dimensional ^15^N-edited NOESY-HSQC (mixing time 100 and 150 ms) and ^13^C-edited NOESY-HSQC (100 ms) spectra. The NOE crosspeaks were picked and quantified by peak-picking algorithm in Aurelia. The NOE peak intensities were converted into upper distance bounds using CALIB module in the torsional angle dynamics program CYANA [69]. Intramolecular NOE distance restraints of CaM and CaNp were primarily deduced from 3D ^15^N- and ^13^C-edited NOESY-HSQC spectra of ^13^C, ^15^N-labeled CaM in complex with unlabeled CaNp, and unlabeled CaM in complex with ^13^C, ^15^N-labeled CaNp sample, respectively. Intermolecular NOE restraints were obtained and confirmed from ^15^N- and ^13^C-edited NOESY-HSQC spectra of both ^13^C^15^N-labeled CaM and CaNp. The backbone dihedral angles (φ and ψ) restraints were derived by chemical shifts of ^13^C^α^, ^13^C^β^, ^13^CO, ^1^H^α^, and ^15^N nuclei using the program TALOS [70,71]. Hydrogen bond restraints were assigned between slowly exchanging amide protons and their respective carbonyl acceptors deduced from the NOE data in combination with the secondary structure information predicted from CSI [72]. Distance restraints between the Ca^2+^ ions and the side chain oxygen atoms of Asp and Asn residues of CaM were obtained from PDB (PDB ID 2BBM). Initial structures were generated by the automated module, NOEASSIGN in CYANA. These NOE assignments were carefully confirmed and erroneous ones corrected through examination of spectra. Additional NOE were then added manually before recalculation of structures by CYANA. The final 100 structures were calculated, and the 20 conformers with the lowest target function values selected and further refined by the restrained simulated annealing and energy minimization algorithms in CNS 1.2 [73,74]. Graphical visualization and analyses of the structures were carried out with the programs MOLMOL [75] and PyMOL (DeLano Scientific, Palo Alto, CA, USA). The geometric and stereo-chemical quality of the ensemble of structures was validated by PROCHECK-NMR [76].

### 3.7. Data Deposition

^1^H, ^13^C, and ^15^N chemical shift assignments were deposited in the Biological Magnetic Resonance Data Bank (http://www.bmrb.wisc.edu) under accession code 15624. The coordinates of the final ensemble of 20 structures and the NMR restraints used for structural determination were deposited in the PDB under accession code 2JZI.

### 3.8. Chemical Shift Deviation of CaM upon Association with CaNp

Chemical shift perturbation map was used to identify the regions of CaM binding site. A single quantity used as normalized chemical shift perturbation of each residue (Δ*δ_residue_*) was expressed as:

(1) $$\Delta \delta_{residue}={\left\{\frac{1}{2}[{\left(\Delta \delta_{HN}\right)}^{2}+{\left(\frac{\Delta \delta_{N}}{5}\right)}^{2}]\right\}}^{\frac{1}{2}}$$

Δ*δ_HN_*, and Δ*δ_N_* represent the chemical shift difference of CaM upon association with CaNp for amide proton and amide nitrogen nucleus, respectively.

### 3.9. Analysis of the Size and Molecular Weight of CaM/CaNp Complex by Dynamic Light Scattering (DLS)

DLS measurements were carried out on a Zetasizer Nano-ZS instrument (Malvern instruments, Malvern, UK) equipped with a He/Ne laser running at 633 nm (4.0 mW) as the light source. Time dependent fluctuations in the scattered light was detected by an avalanche photodiode and then passed to a digital correlator with a sampling time range from 0.5 µs to 80 s. The autocorrelation function of the scattered light intensity data was then used to obtain the translational diffusion coefficient (D). The hydrodynamic radius (R_H_) and the size distribution of the molecules were derived from the Stokes-Einstein equation using the manufacturer’s Nano software. Double logarithmic calibration curve of R_H_ versus molecular weight (M_r_) of standard globular proteins was obtained, and used to estimate the molecular weight of the molecules. Standard protein samples used to obtain the calibration curve included bovine serum albumin (M_r_ 67 kDa), CaM (M_r_ 16.7 kDa), lysozyme (M_r_ 14.7 kDa), and ubiquitin (M_r_ 8.4 kDa). Samples (1 mg/mL protein in 5 mM CaCl_2_ and 25 mM Tris, pH 8.0) were filtered through pre-rinsed filter and a minimum 5 measurements per sample were made. The molecular weight of CaM/CaNp complex was estimated by linear interpolation of the calibration curve.

### 3.10. Secondary Structure Changes of CaM by Circular Dichroism Spectroscopy

CD spectra were recorded on a 715 CD spectrometer (Jasco, Tokyo, Japan). CD spectra were collected using a cylindrical quartz cuvette with a 1 mm path-length. The step resolution was 0.2 nm with 1.0 nm bandwidth at a scan speed of 50 nm/min. Each CD spectrum was averaged over 16 measurements and corrected for the appropriate buffer baseline. The titration experiment was performed in which various amounts of CaNp were added to a fixed amount CaM. All spectra are presented as the molar CD absorption coefficient (Δε_M_) using the molar concentration of CaM to facilitate direct comparison of free CaM and CaM/peptide complex. The difference between the CD spectra in the presence and the absence of the peptide was used to estimate the secondary structure of the peptide upon complex formation. Sample concentrations of CaM were 20 µM, CaNp were ranged from 1 to 30 µM.

## 4. Conclusions

Unraveling the diverse activation mechanisms and target recognition of CaM-dependent processes has received extensive attention in the past. The rules underlying the recognition of CaM with manifold target sequences have been proposed extensively and revised many times in literature. In this paper, we present the spectroscopic studies on CaM with the target sequence of CaN. Our results demonstrated that a new binding family 1–18 was found with major anchor residues Ile^396^, and Leu^413^ to allocate the largest space between two domains of CaM. Both the hydrophobic and electrostatic distribution determines the relative binding orientation of CaM-binding domain peptides. The relative orientation of CaNp to CaM is similar to the CaMKK peptide in the 1–16 binding mode, in which the N- and C-terminal hydrophobic anchors of target peptide were engulfed in the hydrophobic pockets of the N- and C-domain of CaM, respectively. In order to accommodate the longest spacing in the sequence, CaNp folds up into a V-shape helix with a kink in the middle. Based on our findings, we suggest that the hydrophobic interactions between the Ca^2+^-saturated C-domain and C-terminal half of the target sequence may provide driving force for the initial recognition. Subsequently folding up the target sequence and readjusting in the helix I of the N-domain allow the two domains of CaM to grab the target sequence. The electrostatic repulsion between CaM/CaNp complex and AID may result in the displacement of AID from active site to full activation. Our findings serve as a structural model for the recognition of CaM by CaN, and provide new insights in the mechanism of activation of CaN.

## Figures and Tables

**Table 1:** Structural statistics of 20 lowest-energy structures of CaM/CaNp complex.

Restraints	Number
Intra-residue NOEs (|i-j| = 0)	702
Sequential NOEs (|i-j|= 1)	643
Medium range NOEs (2 ≤|i-j| ≤ 5)	676
Long range NOEs (|i-j| > 5)	
Intra molecule NOEs	342
Inter molecule NOEs	128
Total NOEs	2491
Dihedral angle restraints	
CaM	252 (φ = 124,ψ = 122,χ = 6)
CaNp	42 (φ = 21, ψ = 21)
Hydrogen Bonds	
CaM	46 (representing 23 H-bonds)
CaNp	26 (representing 13 H-bonds)
**Restraints violations**	
Distance violations	0
van der Waals violations	0
Angle violations	0
**Ramachandran analysis**	
Residues in most favored regions (%)	92.6
Residues in additional allowed regions (%)	5.6
Residues in generously allowed regions (%)	1.9
Residues in disallowed regions (%)	0.0
**RMSD from mean structure (Å)**	
Backbone	0.66 ± 0.24
Heavy atoms	1.14 ± 0.28

## Supplementary Materials

Supplementary Materials are available online.
