# Supplementary material for: The Recognition of Calmodulin to the Target Sequence of Calcineurin—A Novel Binding Mode
Source: Molecules. 2017 Sep 21;22(10):1584. doi: 10.3390/molecules22101584 (PMC6151454; doi:10.3390/molecules22101584)
Supplement: Supplementary file 1 [file molecules-22-01584-s001.pdf]

Table S1. Chemical shift deviation of free CaM and bound form CaM

Residue	free CaM		bound form CaM		$\Delta\delta_{residue}$
	Chemical Shift (ppm)				
	N	NH	N	NH	
Q3	119.950	8.352	120.107	8.317	0.033
L4	123.158	8.286	123.389	8.223	0.055
T5	113.137	8.686	113.163	8.687	0.004
E6	120.682	9.027	120.459	8.968	0.052
E7	119.583	8.699	119.873	8.575	0.097
Q8	120.213	7.755	119.052	7.707	0.168
I9	119.658	8.368	119.873	8.429	0.053
A10	121.444	8.037	122.129	8.071	0.100
E11	119.539	7.853	118.789	7.965	0.132
F12	120.125	8.588	118.965	8.452	0.190
K13	123.480	9.191	124.004	9.244	0.083
E14	120.580	7.860	119.492	8.006	0.185
A15	122.660	8.030	120.986	7.742	0.312
F16	119.261	8.811	119.052	8.646	0.120
S17	112.683	7.978	113.896	8.200	0.233
L18	121.122	7.460	120.605	7.202	0.197
F19	114.997	7.296	112.518	6.932	0.435
D20	117.473	7.899	116.035	7.425	0.392
K21	124.784	7.702	124.063	7.654	0.107
D22	114.177	8.162	114.112	8.161	0.009
G23	109.415	7.716	109.207	7.625	0.071
D24	120.873	8.437	120.957	8.470	0.026
G25	113.371	10.654	112.724	10.505	0.140
T26	113.093	8.201	112.518	8.235	0.085
I27	127.304	9.814	126.905	9.807	0.057
T28	116.741	8.529	116.562	8.475	0.046
T29	113.093	9.106	112.489	9.203	0.110
K30	120.873	7.729	121.104	7.595	0.100
E31	121.561	7.689	122.070	7.689	0.072
L32	120.536	8.719	119.785	8.610	0.131
G33	105.679	8.686	105.721	8.786	0.071
T34	118.074	7.939	118.496	8.053	0.100
V35	122.367	7.735	121.865	7.748	0.072
M36	118.367	8.522	117.294	8.434	0.164

R37	119.158	8.496	119.052	8.675	0.127
S38	119.012	8.037	119.111	7.983	0.041
L39	120.492	7.427	119.463	7.314	0.166
G40	106.793	7.899	106.893	7.871	0.024
Q41	118.558	7.880	118.320	7.854	0.038
N42	116.536	8.680	116.474	8.593	0.062
P43	NA	NA	NA	NA	
T44	112.917	8.712	113.837	9.156	0.340
E45	120.975	8.837	120.627	8.769	0.069
A46	120.726	8.273	120.576	8.182	0.068
E47	118.953	7.716	118.789	7.672	0.039
L48	120.565	8.207	119.433	8.235	0.161
Q49	118.235	8.234	117.887	8.144	0.080
D50	120.037	8.076	120.635	8.059	0.085
M51	119.437	7.998	120.137	8.153	0.148
I52	118.103	7.775	117.324	7.824	0.115
N53	117.810	8.568	117.646	8.511	0.047
E54	116.609	7.604	116.708	7.478	0.090
V55	109.694	7.263	111.845	7.261	0.304
D56	121.473	7.729	120.898	7.801	0.096
A57	131.993	8.476	131.359	8.159	0.241
D58	114.030	8.227	114.306	8.276	0.052
G59	108.492	7.604	108.680	7.601	0.027
N60	119.657	8.371	118.965	8.241	0.134
G61	113.488	10.588	112.958	10.429	0.135
T62	109.064	7.716	108.534	7.678	0.080
I63	124.008	8.929	123.155	8.634	0.241
D64	128.403	8.896	128.927	9.115	0.172
F65	119.100	8.981	118.994	8.939	0.033
P66	NA	NA	NA	NA	
E67	117.459	7.794	117.651	8.111	0.226
F68	123.539	8.804	123.594	8.628	0.125
L69	119.715	8.386	120.166	8.816	0.311
T70	115.320	7.591	115.126	7.795	0.147
M71	121.590	7.761	120.283	7.472	0.276
M72	117.019	8.089	115.917	7.859	0.225
A73	122.059	8.214	121.104	8.053	0.177
R74	116.917	7.584	118.320	7.191	0.341

K75	ND	ND	ND	ND	
M76	118.895	7.761	122.363	8.335	0.637
K77	119.246	7.906	121.572	8.534	0.553
D78	121.678	8.280	122.100	8.554	0.203
T79	114.675	8.109	117.910	8.135	0.458
D80	123.261	8.450	122.686	7.619	0.593
S81	117.034	8.404	116.269	8.323	0.122
E82	122.733	8.549	121.191	8.247	0.305
E83	119.744	8.063	118.701	7.936	0.173
F84	120.521	7.761	117.705	7.947	0.419
I85	121.971	8.057	119.609	7.683	0.426
R86	121.825	8.424	122.129	8.563	0.107
E87	118.631	8.129	117.412	8.258	0.195
A88	122.001	7.978	120.738	8.098	0.198
F89	118.983	8.562	118.379	8.610	0.092
R90	115.701	7.722	115.771	7.824	0.073
V91	118.426	7.532	117.412	7.496	0.146
F92	116.287	7.447	116.503	7.455	0.031
D93	116.902	7.834	116.650	7.818	0.037
K94	126.030	7.748	126.495	7.777	0.069
D95	114.280	8.266	114.042	8.153	0.087
G96	109.371	7.807	109.354	7.824	0.012
N97	119.657	8.404	119.404	8.323	0.068
G98	113.078	10.667	112.853	10.696	0.038
Y99	115.994	7.657	115.859	7.642	0.022
I100	127.495	10.155	127.110	10.089	0.072
S101	123.979	9.027	124.034	9.009	0.015
A102	123.100	9.224	123.125	9.262	0.027
A103	118.499	8.240	118.320	8.194	0.041
E104	119.876	7.873	119.990	7.890	0.020
L105	120.975	8.614	120.254	8.587	0.104
R106	117.620	8.594	117.587	8.628	0.024
H107	118.983	8.175	119.287	8.077	0.082
V108	118.968	7.978	118.877	8.194	0.153
M109	116.507	8.293	115.654	8.288	0.121
T110	115.159	8.188	115.361	8.071	0.088
N111	122.323	7.899	122.158	7.654	0.175
L112	118.997	7.866	118.408	7.807	0.093

G113	106.734	7.860	107.655	7.824	0.133
E114	120.785	7.965	119.228	7.848	0.235
K115	123.993	8.509	124.883	8.581	0.136
L116	125.034	8.096	125.440	8.082	0.058
T117	114.426	9.099	114.863	9.198	0.093
D118	121.078	8.876	121.162	8.839	0.029
E119	119.129	8.667	119.111	8.622	0.032
E120	120.521	7.761	120.459	7.771	0.011
V121	121.078	8.109	120.869	8.094	0.031
D122	119.744	8.063	119.404	8.030	0.053
E123	119.407	7.906	119.287	8.123	0.154
M124	119.583	7.821	119.258	7.795	0.050
I125	118.397	7.998	118.173	7.989	0.032
R126	118.484	8.227	118.349	8.376	0.107
E127	116.360	7.925	116.240	7.942	0.021
A128	119.217	7.394	118.994	7.349	0.045
D129	117.679	7.945	117.705	8.018	0.052
I130	128.008	8.457	127.931	8.446	0.013
D131	116.975	8.345	116.767	8.288	0.050
G132	108.639	7.630	108.475	7.578	0.043
D133	120.931	8.378	120.811	8.335	0.035
G134	112.990	10.352	112.870	10.335	0.021
Q135	115.554	7.998	115.390	8.006	0.024
V136	125.561	9.158	125.294	9.080	0.067
N137	129.150	9.598	129.103	9.625	0.020
Y138	118.528	8.450	118.584	8.446	0.008
E139	118.499	8.109	118.701	8.100	0.029
E140	119.920	8.771	119.873	8.792	0.016
F141	124.565	8.949	124.854	8.863	0.073
V142	119.539	8.594	119.521	8.669	0.053
Q143	118.338	7.493	119.580	7.724	0.240
M144	119.437	7.998	119.463	7.924	0.052
M145	115.085	7.912	115.038	7.760	0.108
T146	111.203	7.657	111.053	7.707	0.041
A147	126.411	7.735	127.052	7.689	0.096
K148	125.532	7.696	126.436	7.959	0.226

Table S2. The half-width measurements of the amide protons of free and bound calmodulin from 2-dimensional ^{1}H-^{15}N HSQC spectra

Residue	free CaM			bound form CaM		
	Chemical Shift (ppm)		half-width (Hz)	Chemical Shift (ppm)		half-width (Hz)
	N	NH		N	NH	
L4	123.158	8.286	14.7	123.389	8.223	14.6
T5	113.137	8.686	18.9	113.163	8.687	17.8
K13	123.480	9.191	15.4	124.004	9.244	14.6
L18	121.122	7.460	15.8	120.605	7.202	17.2
F19	114.997	7.296	20.0	112.518	6.932	19.5
K21	124.784	7.702	14.0	124.063	7.654	15.1
G23	109.415	7.716	15.6	109.207	7.625	15.6
T26	113.093	8.201	19.1	112.518	8.235	17.9
I27	127.304	9.814	21.6	126.905	9.807	21.0
T29	113.093	9.106	14.8	112.489	9.203	15.2
G33	105.679	8.686	17.8	105.721	8.786	17.7
G40	106.793	7.899	20.0	106.893	7.871	18.1
T44	112.917	8.712	18.5	113.837	9.156	16.0
V55	109.694	7.263	21.7	111.845	7.261	19.3
A57	131.993	8.476	15.3	131.359	8.159	15.7
G59	108.492	7.604	18.6	108.680	7.601	17.5
G61	113.488	10.588	17.5	112.958	10.429	17.0
T62	109.064	7.716	17.5	108.534	7.678	17.5
I63	124.008	8.929	20.2	123.155	8.634	20.6
D64	128.403	8.896	22.3	128.927	9.115	15.5
F65	119.100	8.981	16.0	118.994	8.939	15.5
K94	126.030	7.748	14.1	126.495	7.777	17.4
G96	109.371	7.807	16.0	109.354	7.824	17.5
I100	127.495	10.155	20.0	127.110	10.089	20.8
S101	123.979	9.027	21.3	124.034	9.009	18.8
A102	123.100	9.224	14.0	123.125	9.262	15.7
G113	106.734	7.860	19.1	107.655	7.824	16.1
K115	123.993	8.509	17.4	124.883	8.581	17.6
L116	125.034	8.096	17.0	125.440	8.082	16.7
T117	114.426	9.099	18.0	114.863	9.198	21.0
I130	128.008	8.457	15.8	127.931	8.446	17.0
G132	108.639	7.630	16.3	108.475	7.578	16.8
G134	112.990	10.352	17.0	112.870	10.335	16.1
V136	125.561	9.158	19.8	125.294	9.080	21.4
N137	129.150	9.598	20.9	129.103	9.625	18.7
F141	124.565	8.949	16.2	124.854	8.863	21.9
T146	111.203	7.657	17.6	111.053	7.707	13.8
A147	126.411	7.735	16.4	127.052	7.689	19.3
K148	125.532	7.696	16.2	126.436	7.959	14.9
Average			17.7			17.4
